# DFUCare: deep learning platform for diabetic foot ulcer detection, analysis, and monitoring

**DOI:** 10.3389/fendo.2024.1386613

**Published:** 2024-09-23

**Authors:** Varun Sendilraj, William Pilcher, Dahim Choi, Aarav Bhasin, Avika Bhadada, Sanjay Kumar Bhadadaa, Manoj Bhasin

**Affiliations:** ^1^ Coulter Department of Biomedical Engineering Emory and Gatech, Atlanta, GA, United States; ^2^ Johns Creek High School, Johns Creek, GA, United States; ^3^ Vivek High School, Chandigarh, India; ^4^ Department of Endocrinology, Postgraduate Institute of Medical Education and Research, Chandigarh, India; ^5^ Aflac Cancer and Blood Disorders Center, Children Healthcare of Atlanta, Atlanta, GA, United States; ^6^ Department of Pediatrics, Emory University, Atlanta, GA, United States; ^7^ Department of Biomedical Informatics, Emory University, Atlanta, GA, United States

**Keywords:** diabetic foot ulcer, machine learning, deep learning - artificial intelligence, wound monitoring, remote health care monitoring

## Abstract

**Introduction:**

Diabetic foot ulcers (DFUs) are a severe complication among diabetic patients, often leading to amputation or even death. Early detection of infection and ischemia is essential for improving healing outcomes, but current diagnostic methods are invasive, time-consuming, and costly. There is a need for non-invasive, efficient, and affordable solutions in diabetic foot care.

**Methods:**

We developed DFUCare, a platform that leverages computer vision and deep learning (DL) algorithms to localize, classify, and analyze DFUs non-invasively. The platform combines CIELAB and YCbCr color space segmentation with a pre-trained YOLOv5s algorithm for wound localization. Additionally, deep-learning models were implemented to classify infection and ischemia in DFUs. The preliminary performance of the platform was tested on wound images acquired using a cell phone.

**Results:**

DFUCare achieved an F1-score of 0.80 and a mean Average Precision (mAP) of 0.861 for wound localization. For infection classification, we obtained a binary accuracy of 79.76%, while ischemic classification reached 94.81% on the validation set. The system successfully measured wound size and performed tissue color and textural analysis for a comparative assessment of macroscopic wound features. In clinical testing, DFUCare localized wounds and predicted infected and ischemic with an error rate of less than 10%, underscoring the strong performance of the platform.

**Discussion:**

DFUCare presents an innovative approach to wound care, offering a cost-effective, remote, and convenient healthcare solution. By enabling non-invasive and accurate analysis of wounds using mobile devices, this platform has the potential to revolutionize diabetic foot care and improve clinical outcomes through early detection of infection and ischemia.

## 1 Introduction

Diabetic foot ulceration (DFU) is a serious complication affecting people with diabetes, with more than half of DFUs at risk of becoming infected. Of these infections, approximately 20% require amputation (1, 2). This is a significant concern as patients who undergo amputation due to DFUs have a high mortality rate, with more than half expected to die within five years (3). Additionally, the financial burden associated with treating and managing DFUs and their complications surpasses that of the top five cancers, with an annual cost exceeding 11 billion dollars in the United States alone (4). As the prevalence of Diabetes Mellitus (DM) continues to rise, DFUs are expected to become an even greater burden for global health systems and may be one of the most expensive diabetes complications (5).

Despite significant improvement in identifying novel therapies for DFU treatment, the early diagnosis of the underlying cause and management of DFU still remains challenging. Impaired DFU healing is complex pathogenesis driven by multiple factors including diabetic foot infections, wound ischemia, exhausted immune system, and poor glycemic control (6–8). DFU management requires infection and ischemia evaluation at multiple time points for better management, which is currently limited due to its invasive nature. This problem is more aggravated in the rural areas of the country due to limited access to DFU wound centers and clinical experts. Therefore, there is an unfulfilled clinical need for non-invasive tools for the analysis of wound infection as well as ischemia detection, two key factors associated with impaired wound healing.

In recent years, DL algorithms have demonstrated great potential in the detection and diagnosis of diseases, particularly in medical imaging, radiology, and pathology (9–11). This has led to the emergence of DL image analysis as an assistive tool, supporting clinicians with decision-making procedures and enhancing the efficiency and accuracy of disease diagnosis and treatment (12). DL has also shown promising results in the classification and localization of DFUs. It achieved high accuracies in ischemia and infection classification, ranging from 87.5% to 95.4% and 73% to 93.5%, respectively (13–16). Furthermore, researchers have made significant progress in DFU localization, with Mean Average Precision (mAP) values between 0.5782 and 0.6940, and F1-scores between 0.6612 and 0.7434 (17, 18).

Despite these advancements, many of these tools are still in the early stages of development and lack automated analysis capabilities for predicting infections, ischemia, and other physical features crucial for DFU wound management. Additionally, current wound analysis platforms rely on proprietary hardware attachments, such as thermal scanners (e.g., SmartMat by Pod Metrics), 3D scanners using structured light or lasers (e.g., Insight 3D by Ekare.ai and Ray 1 by Swift Medical), and Optical Coherence Tomography (OCT) for visualizing and quantifying microvascular structures related to DFU formation (19, 20). The need for these specialized attachments may restrict the access to DFU management among the general population.

To address these limitations, it is essential to develop a non-invasive and automated tool that can comprehensively analyze wound tissues, even in resource-limited areas. This study aims to investigate this issue by introducing the DFUCare, a novel approach that enables the comprehensive analysis of wounds through images captured using standard phone hardware. DFUCare incorporates key components such as wound region detection models, infection and ischemia classification, size measurement, and traditional color and textural analysis. DFUCare’s non-invasive nature, coupled with its automated analysis, empowers clinicians to manage infections, ischemia, and other critical physical features more effectively, ultimately enhancing DFU wound management.

## 2 Materials and methods

**Figure 1:**
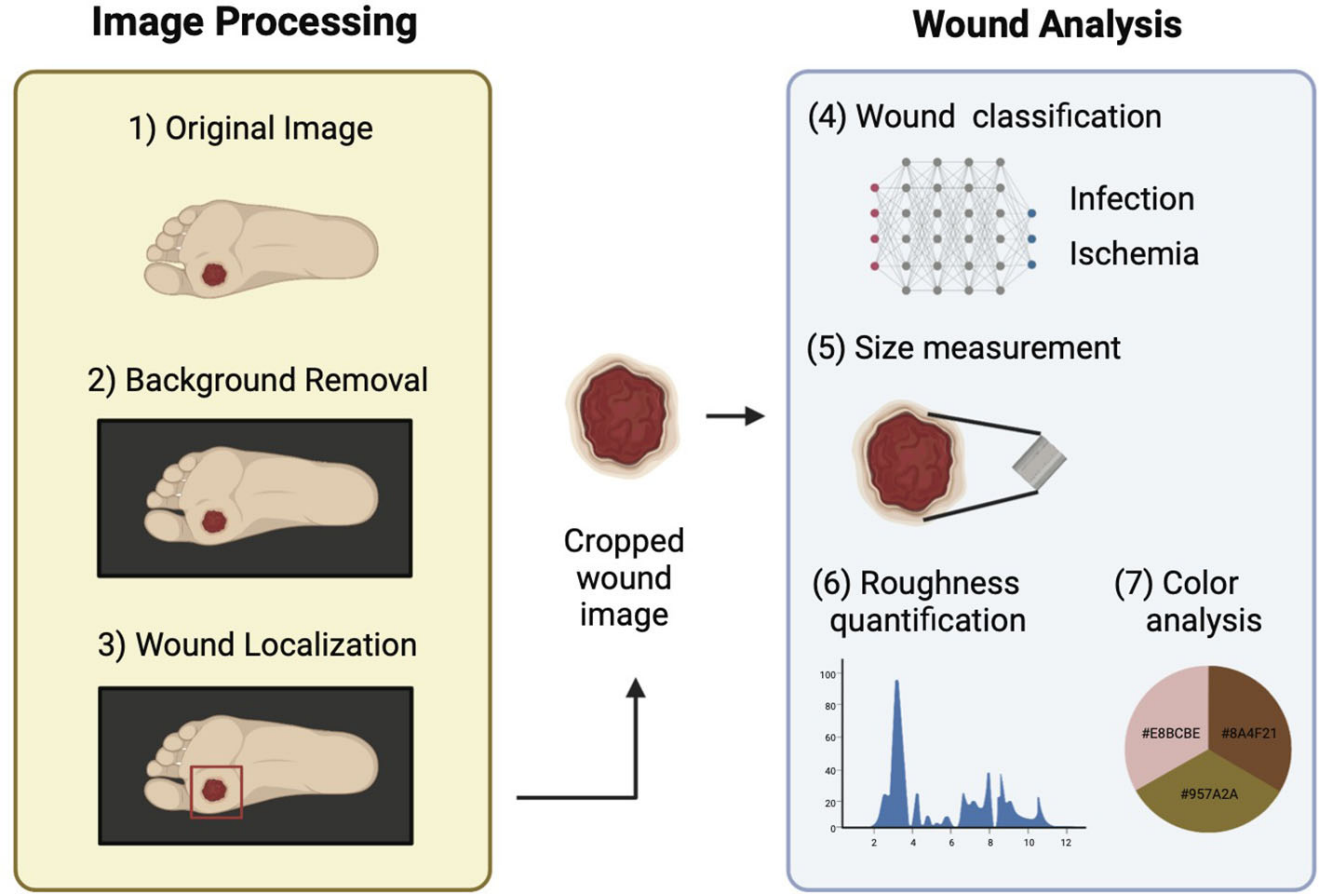
Schematic overview of DFUCare platform comprises two main components: 1) Image preprocessing that includes background removal and wound localization. 2) Image analysis which contains wound classification, size measurement, roughness quantification, color analysis.

This section provides a detailed description of the datasets used in the study and different components of DFUCare. The platform involves localizing, cropping, and classifying the wound, and analyzing macroscopic features such as size, color, and texture extracted from the cropped wound image to determine their association with infection and ischemia status (**Figure 1**).

### 2.1 DFU datasets

#### 2.1.1 DFUC2020

The goal of the Diabetic Foot Ulcer Competition 2020 (DFUC 2020) dataset was to improve the accuracy of DFU detection in real-world settings (18). The dataset consisted of foot images with DFUs collected from Lancashire Teaching Hospitals. The images were captured using three digital cameras (Kodak DX4530, Nikon D3300 and Nikon COOLPIX P100), and close-ups of the foot were taken without zoom or macro functions. The dataset comprised of 4,000 images, with 2,000 used for training and 2000 for testing. The images were acquired during regular patient appointments, resulting in variability in factors such as distance, angle, lighting, and the presence of background objects. The dataset included cases with multiple DFUs, different stages of healing, partial foot visibility, and foot deformities. The dataset also featured cases with time stamps, rulers, and partial blurring or obfuscation of wounds. The images were annotated by healthcare professionals, indicating the ulcer location using bounding boxes.

#### 2.1.2 DFUC 2021

Diabetic Foot Ulcer Competition 2021 (DFUC 2021) dataset was developed to enhance the accuracy of DFU classification in clinical settings (21). Collected during patient visits at Lancashire Teaching Hospitals, the dataset features 15,683 foot images captured with three different camera models. To ensure consistency, close-up photographs of the entire foot were taken at a distance of 30-40 cm, maintaining a parallel orientation to the ulcer plane and using adequate room lighting to achieve consistent colors. The dataset includes annotations by a podiatrist and a consultant physician for ulcer location, ischemia, and infection status. Data curation involved cropping DFU regions and applying natural data augmentations.

### 2.2 Wound image preprocessing

**Figure 2:**
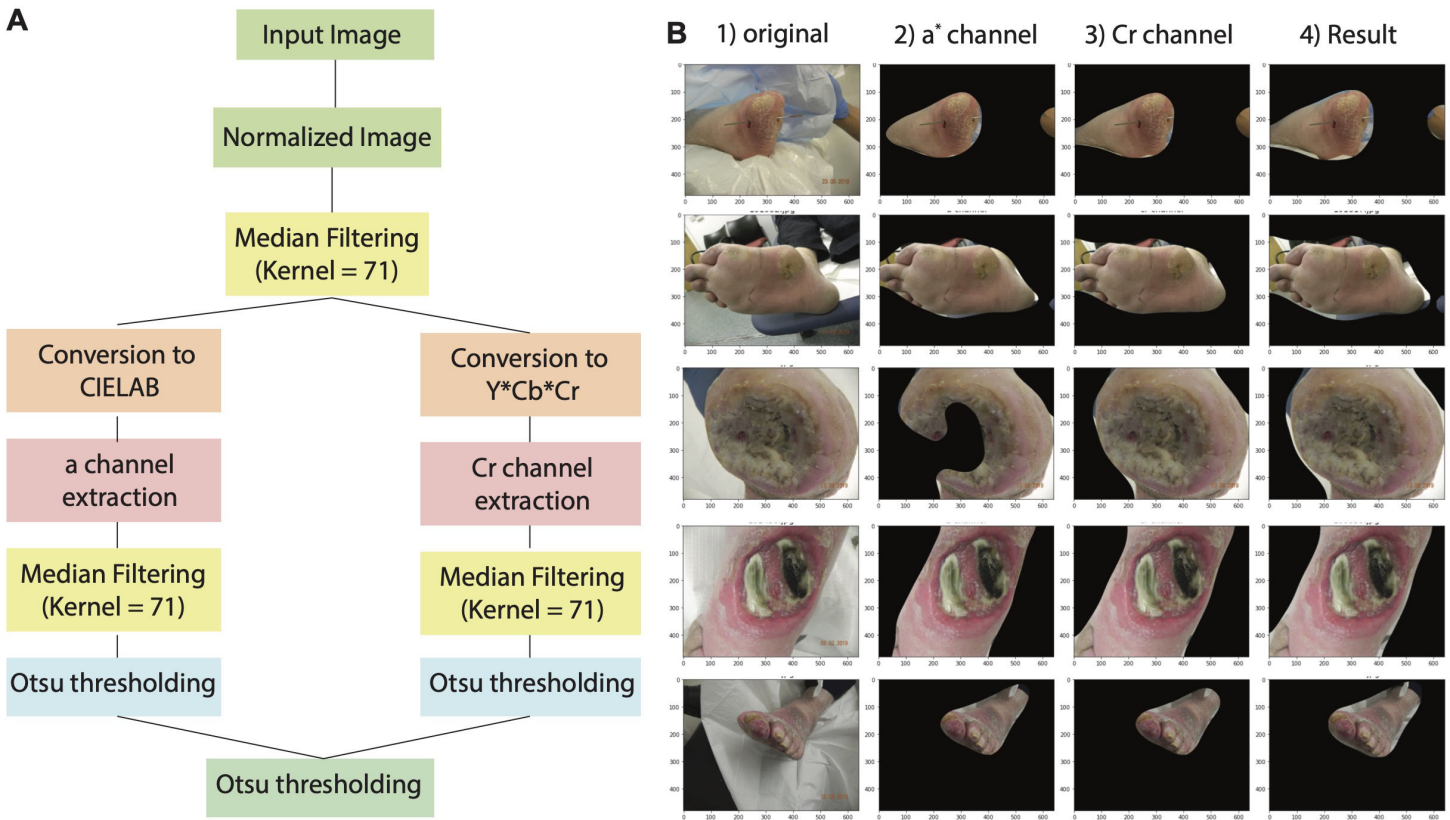
Overview of wound image normalization and background removal approach. **(A)** Diagram illustrating the workflow of image normalization and background process. **(B)** Example showing segmentation results based on a* channel, Cr channel, and the merge of both color space. Area marked by red dotted line demonstrates false positive region and yellow dotted line indicates false negative region.

To optimize the performance of wound detection model, a comprehensive image preprocessing pipeline with the primary objective of removing background regions, in the wound images was applied (**Figure 2A**). Before background removal, min-max image normalization was applied to ensure the comparability of wound images across different samples. This technique rescaled the pixel intensities of each image to a specific range, between 0 and 1. By normalizing the pixel intensities through subtracting the minimum value and dividing by the range of pixel values, consistent intensity levels across all samples were achieved, accounting for variations in camera resolution and lighting conditions.

To accurately distinguish between the skin and background regions in the wound images, we implemented a colorspace thresholding approach. Extensive research has demonstrated the effectiveness of the Cr channel in the YCRCB colorspace, as well as the a* channel in the CIELAB colorspace, for precise skin-to-background segmentation (22). Leveraging this knowledge, we generated a binary mask by applying Otsu’s thresholding technique to the Cr channel in the CIELAB colorspace and the CR channel in the YCbCr colorspace. This binary mask was applied on the original wound image to separate the foreground from the background skin. In addition, median filtering was incorporated to refine the binary mask obtained from the thresholding process and minimize background region inconsistencies (**Figure 2B**). This technique replaced each pixel with the median value of its neighboring pixels, resulting in the removal of isolated background region pixels while preserving the overall structure of the mask. By incorporating this multi-step approach, our platform achieved a significant reduction in background region in the wound images.

### 2.3 Wound detection and localization

DL-based object localization models, such as the YOLO series, have consistently demonstrated exceptional speed and accuracy in detecting objects. In particular, YOLOv5 exhibits improved learning capabilities compared to its predecessors and utilizes the BottleneckSCP technique to extract hierarchical features with reduced computational complexity (23).

For our study, we employed the YOLOv5s model, pretrained on the COCO dataset, and fine-tuned it on the DFUC 2020 dataset to enhance model convergence. The DFUC 2020 dataset was divided into a training set (n=1800) and a test set (n=200), and a 10-fold cross-validation technique was applied, training each fold for 30 epochs.

To address the limited number of wound images in the dataset, we employed data augmentation techniques. These included adjusting the hue, saturation, and value (HSV) of the images, as well as utilizing translation, scaling, flipping, and mosaic techniques. This augmented dataset improved model performance and generalization.

Additionally, the YOLOv5s model employs a stochastic gradient descent (SGD) optimizer with an initial learning rate of 0.01 (24). The chosen learning rate ensures a balance between convergence speed and accuracy, allowing the model to effectively optimize its performance in detecting wounds.

To improve the localization accuracy of the model and reduce generalization error, the weights were tuned to achieve the highest mAP and Intersection over Union (IoU) scores within the range of 0.5 to 0.95. A 10-fold cross-validation process was performed and the weights that achieved the best mAP and IoU scores were aggregated. This ensures that the selected weights yield improved localization performance on the DFUs even for unseen wound images beyond the training set.

### 2.4 Automated classification of infection and ischemia in wound images

To classify the detected wound images into four categories: i) infection, ii) ischemia, iii) both infection and ischemia, and iv) neither infection nor ischemia, both a classical machine learning pipeline trained on hand-crafted image features and a DL pipeline were developed. The inclusion of the classical machine learning approach facilitates the extraction of interpretable wound features, ensuring transparency and practicality in medical application. The DL-based approach automatically learns complex patterns and hierarchical representations from wound images, capturing subtle features and nuances not easily discernible through traditional hand-crafted feature extraction, increasing the model performance.

### 2.5 Deep learning-based classification of DFU

**Figure 3:**
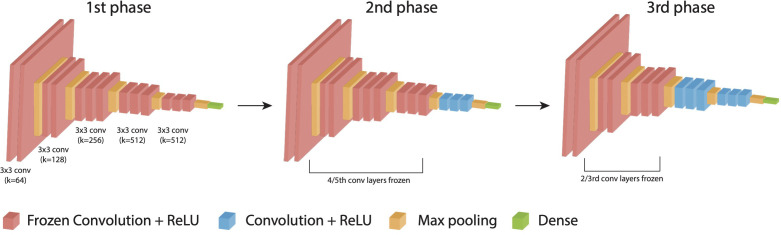
DL-based DFU classification model and training. Training strategy composed of three phases down with an example of VGG 16 architecture.

To determine the CNN architecture that achieved the highest DFU classification reliability, we chose four most popular pre-trained ImageNet models (Resnet50v2, VGG16, InceptionResNetV2, and DenseNet121) and trained into three phases of 20 epochs each (25–28). For each model architecture, variants were trained with and without the addition of an additional dense layer between the last convolutional layer output, and the output node. Approximately 20% of images from the training dataset were held out for validation (1,156 images). To prevent overfitting and improve the performance of the DL models, image augmentation techniques including random rotations, flips, and shifts in brightness to each image in each epoch. Additionally, binary cross-entropy was used as a loss function to update the weights in each iteration. We evaluated the performance of the algorithms using multiple metrics, including binary accuracy, area under the curve (Area under the ROC (Receiver Operating characteristic curve) Curve), precision, and recall. All four models as-is with single output node and the same four models with a trainable dense layer after the last convolutional layer were trained on the binary classification tasks for either the presence of infection or ischemia. An output node following the last convolutional layer with a sigmoid activation function was used to give the binary classification result. Models were trained by three phases of 20 epochs each: 1) All weights for convolution layers were frozen and optimized by Adam with learning rate of 3e-4. 2) 4/5ths of the convolutional layers were frozen and RMSprop with learning rate of 1e-5 was used for optimization. 3) 2/3rds of the layers remained frozen and optimized with decayed learning rate of 1e-6 on binary cross entropy loss in Tensorflow2 (**Figure 3**) (29).

Due to imbalances in the number of ischemia images present (179 of the 4,799 images), ischemic models were trained both on the dataset as-is, and with ischemia-only and ischemic and infected images upsampled by a factor of six with random augmentations. This duplication brings the number of positive ischemic cases (662) in line with the number of negative ischemic cases (4,137). No modifications were made to the validation dataset.

The binary classification results were converted to a four-way classification result through the following formulas:


(1) P(none)=(1−P(Inf)) * (1−P(Isch))

(2) $$P\left(Inf_{Only}\right)=P\left(Inf\right)\;*\;\left(1-P\left(Isch\right)\right)$$

(3) $$P\left(Isch_{Only}\right)=\left(1-P\left(Inf\right)\right)\;*\;P\left(Isch\right)$$

(4) P(Both)=P(Inf) * P(Isch)

Where P(Inf) is the output of the binary infection model, and P(Isch) is the prediction of the binary ischemia model. Four classification accuracy, F1-Score, and AUC were assessed on the training, validation, and test dataset by combining each network architecture’s best infection or ischemia models.

### 2.6 Handcrafted features extraction and classical machine learning-based DFU classification

The classical machine learning algorithm for wound classification was a comprehensive approach that incorporates six visual analysis methods to extract features from wound images (30). The algorithm computed the distribution of CIELAB color space channels, the Gray Level Co-occurrence Matrix (GLCM) for the full image, distribution of GLCM metrics for 64x64 pixel patches across an image, Local Binary Patterns (LBP), Local Phase Quantification (LPQ), and Gabor filter to extract a mixture of color and textural features (**Supplementary Figure S1**). These handcrafted features are used to train classical models including a non-linear SVM model using a Radial Basis Function (RBF) kernel, Gradboost [100 tress with depth of 3 either on raw features or after applying Principal Component Analysis (PCA)], XGBoost (100 tress with depth of 3, raw features or after PCA), and multilayer perceptron (MLP) with three layers to classify infected vs non-infected or ischemic vs non-ischemic DFUs (31–33). The algorithm was trained on a dataset of 4799 images using 5-fold cross-validation to select the optimal number of PC to use, and additionally tested on the held-out validation set (1,156 images). Two binary classifiers identifying infection and ischemia respectively and multi-classifier with four categories were developed and evaluated using F1-score, precision, recall, and accuracy.

### 2.7 Wound characterization and analysis

#### 2.7.1 Wound size measurement

**Figure 4:**
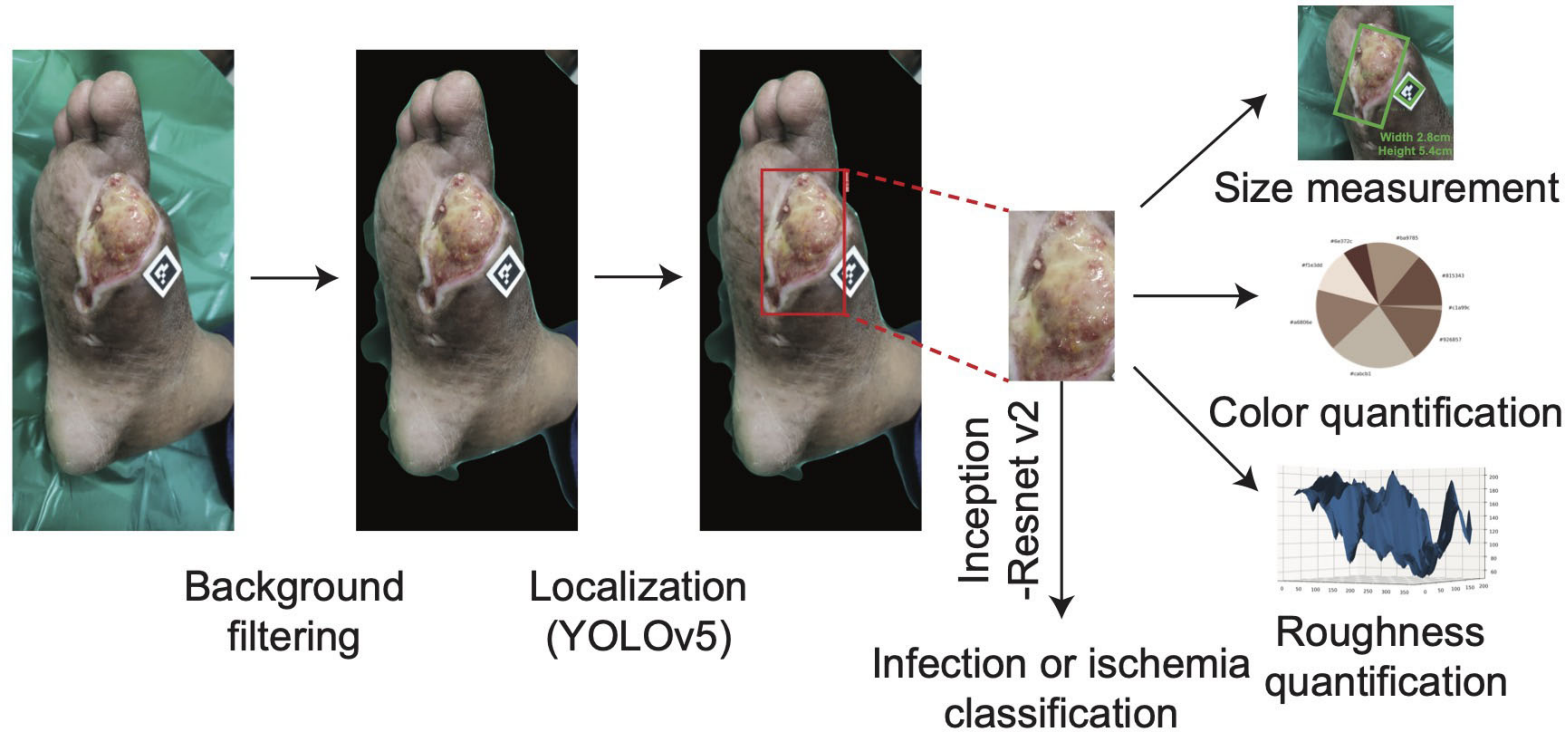
The overall process of DFUCare platform with an example of patient data from PGIMER, A DFU image goes through background filter and YOLO v5. The detected wound region is cropped then undergoes Inception-Resnet v2 to classify the status of wound (infected or ischemic) and is used to measure the size of wound and analyze color composition and roughness.

To determine the surface area of the wound with a camera, DFUCare utilized a 1.3 cm by 1.3 cm ArUco marker placed near the wound along with the Open-cv library to calculate a “pixel to metric” ratio based on the predefined size of the marker. This allows for the conversion of pixel size to a numerical measurement in centimeters (**Figure 4**). This provided the width and height of the wound region using the size of the bounding box from the wound localization.

#### 2.7.2 Color analysis of the wounds

Studies have indicated that ulcers may exhibit varying colors depending on their etiology and stage of healing. Wounds with an overlying layer of black eschar may transition through various colors as part of the healing process, changing from black to yellow, then to red, and eventually to granular red indicating tissue regeneration. While red or yellow hues may sometimes be associated with infection, it is important to note that darker tones, such as black eschar, can also indicate infection or ischemia, especially as the wound progresses through different stages of healing. (30, 34, 35). To incorporate this, DFUCare employed unsupervised K-means clustering to analyze and determine the relative percentage of the seven major colors present in the localized DFU images, providing valuable insights to the clinician for tissue analysis (**Figure 4**). The DFUCare color analysis tool enables physicians to conduct a proper analysis of the coloration of DFU’s by determining the relative percentages of each color present in the wound.

#### 2.7.3 Texture analysis of the wounds

The progression of wound healing can be observed through changes in the wound surface’s texture. A smooth surface is indicative of proper healing as new tissue forms and the wound contracts. Conversely, the presence of roughness may suggest the potential for infection or a delay in tissue regeneration. Furthermore, the accumulation of necrotic tissue, also known as eschar, can contribute to roughness and impede healing. To obtain the roughness values, a two-dimensional grayscale image of the wound surface is transformed into a three-dimensional representation with a height map projection using the Numpy and Scipy libraries. After applying a Gaussian filter to minimize image noise, the roughness can be calculated by analyzing the “bumps” or variations of the surface of the three-dimensional projection. This allowed a graphical representation of the roughness as well as a numerical measurement.

### 2.8 Pilot study for determining the performance of DFUCare algorithm

To test the performance of the DFUCare algorithm, we performed a pilot study in collaboration with the Postgraduate Institute of Medical Education and Research (PGIMER), in Chandigarh, India. Wound images were obtained as part of a routine visit to the foot care lab of the endocrinology clinic at PGIMER. The infection and ischemia status of wounds were determined by a physician at the foot care lab of PGIMER with the help of standard wound culture and wound characteristics. Ischemia was evaluated based on vascular status assessments, including the absence of pulses, Ankle-Brachial Index (ABI), and patterns observed on color Doppler. The wound images with the ArUco marker placed adjacent to the wound were acquired using an iPhone X camera. In addition to wound images, de-identified patient demographics, infection status, ischemia status, and manual wound size (rounded to the nearest whole number) were also collected.

## 3 Results

In this section, we begin by conducting a detailed analysis of the results obtained from each of the individual models on both the DFUC2020 and DFUC2021 datasets. Additionally, we conducted a pilot study in collaboration with the PGIMER, in which the end-to-end DFUCare was tested.

### 3.1 DL model enabled wound localization from healthy skin with high precision

**Figure 5:**
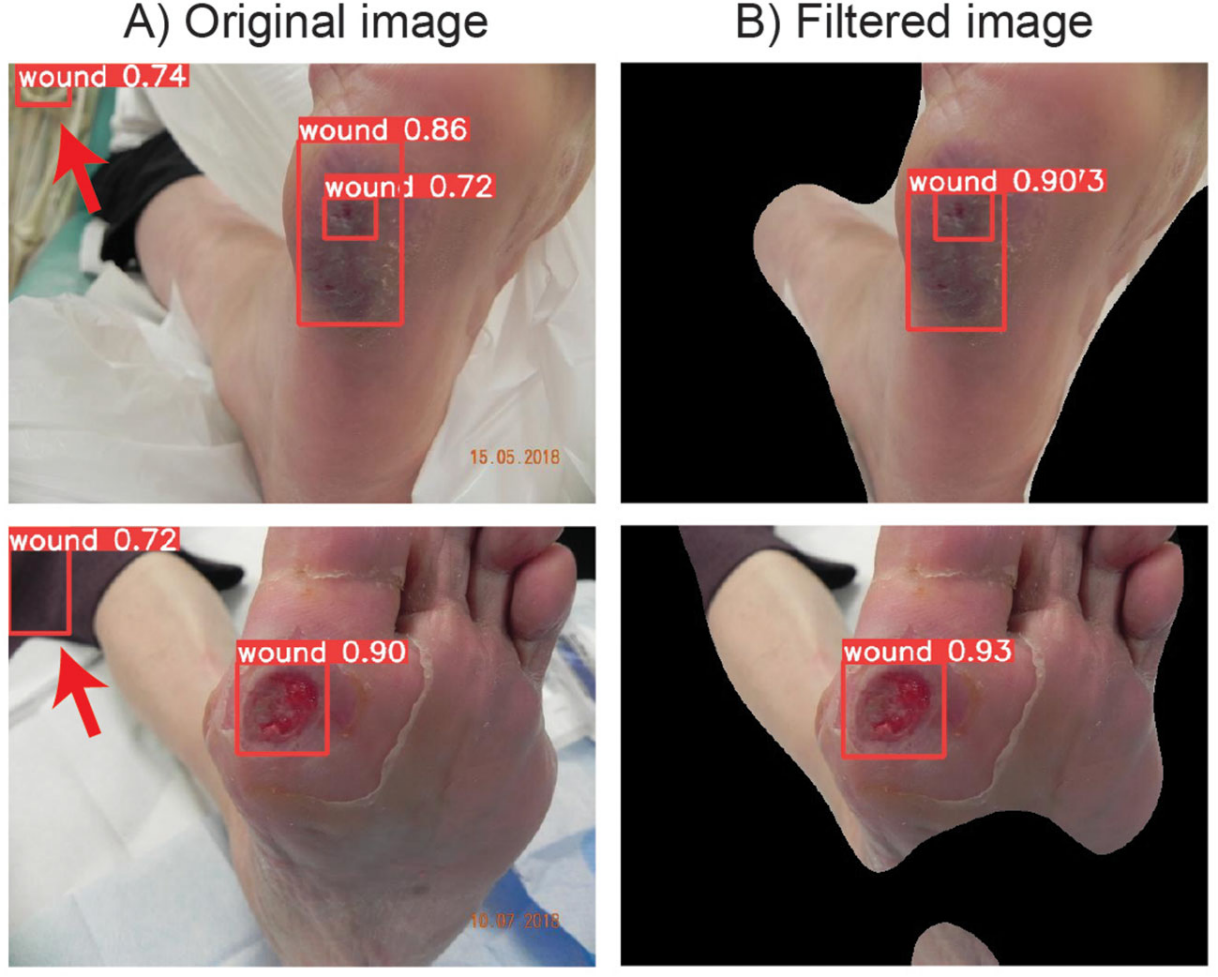
DFUC 2020 examples showing the improvement of wound localization by preventing false positive cases through binary mask filtering. **(A)** Left column shows the wound detection results by YOLOv5s when original images are used. **(B)** Right column shows the detection wound area after iamge preprocessing with enhanced peroformance.

As an initial step of the DFUCare, we developed a wound region detection module using the YOLOv5s model trained on the DFUC2020 dataset. This algorithm achieved an F1-score of 0.78 and a mAP of 0.847 on the test set (**Supplementary Figure S2A**). However, upon further analysis on the incorrectly localized test cases, we observed detection of false positives on the image background (**Figure 5A**).

To prevent false positives, image preprocessing pipeline removing background images was developed. The performance of the image preprocessing pipeline was evaluated using a subset of 100 randomly chosen images from the DFUC2020 dataset. The performance of the workflow was evaluated through manual analysis of the resulting images, specifically by determining if the wound region was unobstructed/visible after background removal was applied. The implemented filtering workflow has shown 97% accuracy in segmenting the foreground from the background for downstream analysis.

Applying this preprocessing step before the wound localization algorithm, performance increase of F1-score of 0.80 and mAP of 0.861 has been observed (**Supplementary Figure S2B**). These results demonstrate the effectiveness of the developed module in accurately detecting and localizing wounds. Additionally, the use of the image preprocessing pipeline further improves the performance of the algorithm by reducing the instances of false positives (**Figure 5B**), highlighting the importance of background region removal in enhancing the precision of wound localization.

### 3.2 Wound infection and ischemia classification

To explore the potential of DL models to determine clinical information from DFU wound images, we conducted extensive training of multiple CNN models using the DFUC2021 dataset. The DFUs detected from the wound localization module were classified on a scale of 0-1, where values below 0.5 indicated non-infected wound, whereas values between 0.5-1, infected wound. Among the tested DL algorithms, the Resnet50v2 model with additional dense layer before output node obtained the best binary classification accuracy, AUC, and precision for infection, and DenseNet121 for ischemia classification, respectively, on the validation set. The DL approach achieved binary classification accuracies of up to 79.76% and 94.81%, for infection and ischemia (**Supplementary Table S1**, **Supplementary Figures S3A, B**, **S4A, B**). In the four-way classification results, the combination of InceptionResnetv2 for infection classification and DenseNet121 with additional dense layer for ischemia classification earned the best F1 score (**Supplementary Figure S4C**). Resnet50v2 demonstrated the highest recall for both. In summary, the InceptionResnetv2 model demonstrated the best classification performance among the DL algorithms tested.

As a comparison to the DL approach, classical machine learning approaches were found to have classification accuracies on held out data ranging from 65.7% to 75.8% for infected or non-infected image patches, and 89.4% to 91.6% accuracy for ischemic and non-ischemic wound patches (**Supplementary Table S2**). Out of the classical models, an SVM model with an RBF kernel using the first 128 principal components showed the best performance on both infection and ischemia. Other classical models tended to overfit the training set, and therefore, poorly generalized to held-out data. While the classical models demonstrate the viability of utilizing handcrafted features for classifying small image patches, these findings suggested that the features learned and utilized by the deep learning architectures are better for the task of wound patch classification.

### 3.3 Demonstration of DFUCare

To validate the results of the DFUCare platform, we collaborated with Dr. Bhadada and his team at the PGIMER, and surveyed a total of 10 patients DFUCare’s results, as shown in **Supplementary Figure S5**, were found to be comparable to the physician’s analysis in **Supplementary Table S3**. The trained YOLO v5s model successfully localized all DFUs diagnosed by physicians except one out of ten patients (patient 4), in which two DFUs were detected. In this test case, the larger bounding box captured the overall wound region, and the smaller bounding box captured the open wound in the overall wound image.

In DFU classification, DFUCare was correct for all of twelve wounds except for the case of ischemic classification for patient 4. This discrepancy may be attributed to the presence of moisture in the image, as ischemia is associated with dryness of the wound and surrounding skin.

In terms of DFU size measurement, DFUCare had an average difference of ± 0.2 cm for length and ± 0.3 cm for width, with the longest side being the length. Additionally, the results from the color analysis module align with the wound classification results (with cases of infection including more yellow hues and cases of ischemia with darker hues), justifying the results from the wound classification algorithm.

Overall, these results demonstrated the relative accuracy and practicality of the DFUCare model in clinical environments.

## 4 Discussion

The aim of this study was to develop a non-invasive, automated, and remote solution for detecting and classifying DFUs using DL-based analysis of wound images. Our approach combined various techniques to perform a comprehensive analysis of wound tissues, differentiating from previous studies. Additionally, unlike existing wound analysis platforms that rely on proprietary hardware attachments, DFUCare only requires standard phone hardware, making it an accessible and portable alternative for DFU management.

Our pipeline successfully detected and localized the wound region with an F1-score of 0.80 and mAP of 0.861, classified infections and ischemia with high level accuracy (79.76% and 94.81% respectively), measured wound size, and analyzed wound color and texture.

These results have significant implications for both wound assessment and reducing physician workload. Assistance in the classification of infections and ischemia enables timely interventions, potentially reducing the risk of severe complications such as amputations. Additionally, the non-invasive nature of DFUCare may increase patient compliance with monitoring regimens to improve outcomes. From a clinical perspective, automated wound analysis significantly reduces the time physicians spend on manual wound measurement and assessment, allowing them to focus on more complex aspects of the patient care.

However, we acknowledged the current dataset’s limitations in regard to diversity in age, race, and types of cameras used. To address this issue, we plan to further validate our preliminary results using a prospective set of images collected from DFU patients at Grady Memorial Hospital. This validation will provide insights into the generalizability of DFUCare to all skin tones, as the patient population predominantly consists of underrepresented minority populations.

In situations where camera quality or lighting conditions may impair the performance of the segmentation or infection/ischemia classification algorithm, we intend to incorporate a user override feature. This will allow users to manually adjust the estimated wound location, ensuring accurate subsequent size estimation or tissue color and texture profiling.

To further improve infection classification accuracy, we plan to explore transformer-based models as well as collecting additional data from diverse patients in future studies. Incorporating attention mechanisms, such as self-attention and spatial attention, could enhance the model’s ability to focus on subtle infection features and important wound regions. Additionally, we propose combining computer vision-extracted features (color and texture) with DL-extracted features to improve phenotype prediction performance. This integration has the potential to yield superior biomarkers for infection classification compared to conventional imaging alone.

Furthermore, we plan to incorporate clinical, biological, and epidemiological features alongside macroscopic image features to enhance the accuracy of classifying infection and predicting curability. Collecting patient records and examining the correlation between patient data and DFU development will provide a wide range of information, including clinical factors (age, gender, medical history, comorbidities, and medication usage), biological markers (blood glucose levels, inflammatory markers, and wound-related characteristics), and epidemiological features (environmental factors and lifestyle choices). Integrating these multifaceted factors with DL analysis of macroscopic image features will enable the development of a comprehensive predictive model for DFU outcomes.

## 5 Conclusion

In conclusion, this study presents a promising approach to developing a non-invasive, and automated, platform for monitoring and managing DFU using DL-based analysis of wound images. The advancements resulting from this research endeavor hold the potential to significantly improve patient outcomes by assisting in better wound management and analysis.

## Data Availability

The original contributions presented in the study are included in the article/**Supplementary Material**. Further inquiries can be directed to the corresponding author.

## References

[1] Armstrong DG Boulton AJM Bus SA. Diabetic foot ulcers and their recurrence. N Engl J Med. (2017) 376:2367–75. doi: 10.1056/NEJMra1615439 28614678

[2] Prompers L Huijberts M Apelqvist J Jude E Piaggesi A Bakker K. High prevalence of ischaemia, infection and serious comorbidity in patients with diabetic foot disease in Europe. Baseline results from the Eurodiale study. Diabetologia. (2007) 50:18–25. doi: 10.1007/s00125-006-0491-1 17093942

[3] Armstrong DG Swerdlow MA Armstrong AA Conte MS Padula WV Bus SA. Five year mortality and direct costs of care for people with diabetic foot complications are comparable to cancer. J Foot Ankle Res. (2020) 13:16. doi: 10.1186/s13047-020-00383-2 32209136 PMC7092527

[4] Gordois A Scuffham P Shearer A Oglesby A Tobian JA. The health care costs of diabetic peripheral neuropathy in the US. Diabetes Care. (2003) 26:1790–5. doi: 10.2337/diacare.26.6.1790 12766111

[5] Sen CK. Human wounds and its burden: an updated compendium of estimates. Adv Wound Care (New Rochelle). (2019) 8:39–48. doi: 10.1089/wound.2019.0946 30809421 PMC6389759

[6] Lane KL Abusamaan MS Voss BF Thurber EG Al-Hajri N Gopakumar S. Glycemic control and diabetic foot ulcer outcomes: A systematic review and meta-analysis of observational studies. J Diabetes Complications. (2020) 34:107638. doi: 10.1016/j.jdiacomp.2020.107638 32527671 PMC7721205

[7] Moura J Madureira P Leal EC Fonseca AC Carvalho E. Immune aging in diabetes and its implications in wound healing. Clin Immunol. (2019) 200:43–54. doi: 10.1016/j.clim.2019.02.002 30735729 PMC7322932

[8] O'brien TD. Impaired dermal microvascular reactivity and implications for diabetic wound formation and healing: an evidence review. J Wound Care. (2020) 29:S21–8. doi: 10.12968/jowc.2020.29.Sup9.S21 32924808

[9] Van Der Heijden,AA Abramoff MD Verbraak F Van Hecke,MV Liem A Nijpels G. Validation of automated screening for referable diabetic retinopathy with the IDx-DR device in the Hoorn Diabetes Care System. Acta Ophthalmol. (2018) 96:63–8. doi: 10.1111/aos.2018.96.issue-1 PMC5814834 29178249

[10] Abramoff MD Lou Y Erginay A Clarida W Amelon R Folk JC. Improved automated detection of diabetic retinopathy on a publicly available dataset through integration of deep learning. Invest Ophthalmol Vis Sci. (2016) 57:5200–6. doi: 10.1167/iovs.16-19964 27701631

[11] Janowczyk A Madabhushi A. Deep learning for digital pathology image analysis: A comprehensive tutorial with selected use cases. J Pathol Inform. (2016) 7:29. doi: 10.4103/2153-3539.186902 27563488 PMC4977982

[12] Cui M Zhang DY. Artificial intelligence and computational pathology. Lab Invest. (2021) 101:412–22. doi: 10.1038/s41374-020-00514-0 PMC7811340 33454724

[13] Yap MH Cassidy B Pappachan JM O’shea C Gillespie D Reeves ND. Analysis towards classification of infection and ischaemia of diabetic foot ulcers, in: 2021 IEEE EMBS International Conference on Biomedical and Health Informatics (BHI). IEEE. (2021). pp. 1–4.

[14] Xu Y Han K Zhou Y Wu J Xie X Xiang W. Classification of diabetic foot ulcers using class knowledge banks. Front Bioeng Biotechnol. (2021) 9:811028. doi: 10.3389/fbioe.2021.811028 35295708 PMC8918844

[15] Goyal M Reeves ND Rajbhandari S Ahmad N Wang C Yap MH. Recognition of ischaemia and infection in diabetic foot ulcers: Dataset and techniques. Comput Biol Med. (2020) 117:103616. doi: 10.1016/j.compbiomed.2020.103616 32072964

[16] Wu X Liu R Wen Q Ao B Li K. DFUC2021 dataset classification based on deep semi-supervised learning methods, in: 2022 2nd International Conference on Consumer Electronics and Computer Engineering (ICCECE). Guangzhou, China: IEEE. (2022). pp. 499–502.

[17] Yap MH Hachiuma R Alavi A Brungel R Cassidy B Goyal M. Deep learning in diabetic foot ulcers detection: A comprehensive evaluation. Comput Biol Med. (2021) 135:104596. doi: 10.1016/j.compbiomed.2021.104596 34247133

[18] Cassidy B Reeves ND Pappachan JM Gillespie D O'shea C Rajbhandari S. The DFUC 2020 dataset: analysis towards diabetic foot ulcer detection. touchREV Endocrinol. (2021) 17:5–11. doi: 10.17925/EE.2021.17.1.5 35118441 PMC8320006

[19] Shah A Wollak C Shah JB. Wound measurement techniques: comparing the use of ruler method, 2D imaging and 3D scanner. J Am Coll Clin Wound Spec. (2013) 5:52–7. doi: 10.1016/j.jccw.2015.02.001 PMC4495754 26199893

[20] Argarini R Mclaughlin RA Joseph SZ Naylor LH Carter HH Yeap BB. Optical coherence tomography: a novel imaging approach to visualize and quantify cutaneous microvascular structure and function in patients with diabetes. BMJ Open Diabetes Res Care. (2020) 8(1):e001479. doi: 10.1136/bmjdrc-2020-001479 PMC7451490 32847842

[21] Diabetic Foot Ulcers Grand Challenge (2020). Available online at: https://dfu-challenge.github.io/dfuc2020.html (Accessed June 11, 2023).

[22] Marijanovic D Filko D. A systematic overview of recent methods for non-contact chronic wound analysis. Appl Sciences-Basel. (2020) 10(21):7613. doi: 10.3390/app10217613

[23] Jocher G. ultralytics/yolov5: v3.1 - bug fixes and performance improvements. (2020). doi: 10.5281/zenodo.4154370

[24] Ruder S. An overview of gradient descent optimization algorithms. arXiv preprint arXiv:1609.04747. (2016). doi: 10.48550/arXiv.1609.04747

[25] Simonyan K Zisserman A. Very deep convolutional networks for large-scale image recognition. arXiv preprint arXiv:1409.1556. (2014). doi: 10.48550/arXiv.1409.1556

[26] He KM Zhang XY Ren SQ Sun J. Identity mappings in deep residual networks. Comput Vision - Eccv. (2016) Pt Iv, 9908:630–45. doi: 10.48550/arXiv.1603.05027

[27] Szegedy C Ioffe S Vanhoucke V Alemi AA. Inception-v4, inception-resNet and the impact of residual connections on learning. Thirty-First Aaai Conf Artif Intell. (2017) 31(1):4278–84. doi: 10.1609/aaai.v31i1.11231

[28] Huang G Liu Z Van Der Maaten L Weinberger KQ. Densely connected convolutional networks, in: Proceedings of the IEEE conference on computer vision and pattern recognition, Honolulu, HI, USA: IEEE. (2017). pp. 4700–8.

[29] Kingma DP Ba J. Adam: A method for stochastic optimization. arXiv preprint arXiv:1412.6980. (2014). doi: 10.48550/arXiv.1412.6980

[30] Goldman RJ Salcido R. More than one way to measure a wound: an overview of tools and techniques. Adv skin Wound Care. (2002) 15:236–43. doi: 10.1097/00129334-200209000-00011 12368715

[31] Cortes C Vapnik V. Support-vector networks. Mach Learn. (1995) 20:273–97. doi: 10.1007/BF00994018

[32] Friedman JH. Greedy function approximation: A gradient boosting machine. Ann Stat. (2001) 29:1189–232. doi: 10.1214/aos/1013203451

[33] Chen T Guestrin C. Xgboost: A scalable tree boosting system, in: Proceedings of the 22nd acm sigkdd international conference on knowledge discovery and data mining, San Francisco, CA, USA: ACM. (2016). pp. 785–94.

[34] Leaper D Assadian O Edmiston CE. Approach to chronic wound infections. Br J Dermatol. (2015) 173:351–8. doi: 10.1111/bjd.2015.173.issue-2 25772951

[35] Keast DH Bowering CK Evans AW Mackean GL Burrows C D'souza L. MEASURE: A proposed assessment framework for developing best practice recommendations for wound assessment. Wound Repair Regener. (2004) 12:S1–17. doi: 10.1111/j.1067-1927.2004.0123S1.x 15230830

