# Supplementary figures and images for: DFUCare: deep learning platform for diabetic foot ulcer detection, analysis, and monitoring
Source: Front Endocrinol (Lausanne). 2024 Sep 23;15:1386613. doi: 10.3389/fendo.2024.1386613 (PMC11460545; doi:10.3389/fendo.2024.1386613)

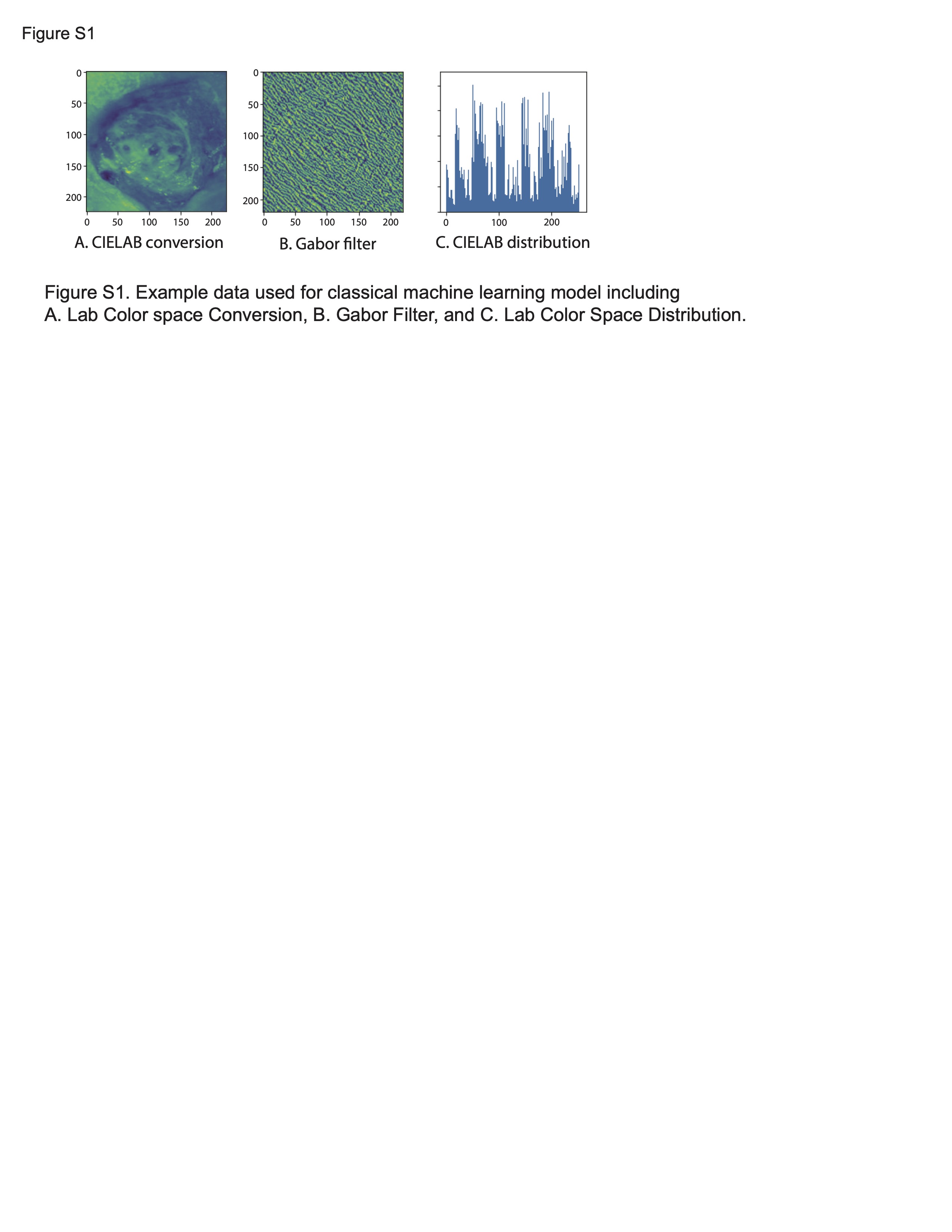

Supplement: Supplementary file 1 [file Image1.jpeg]

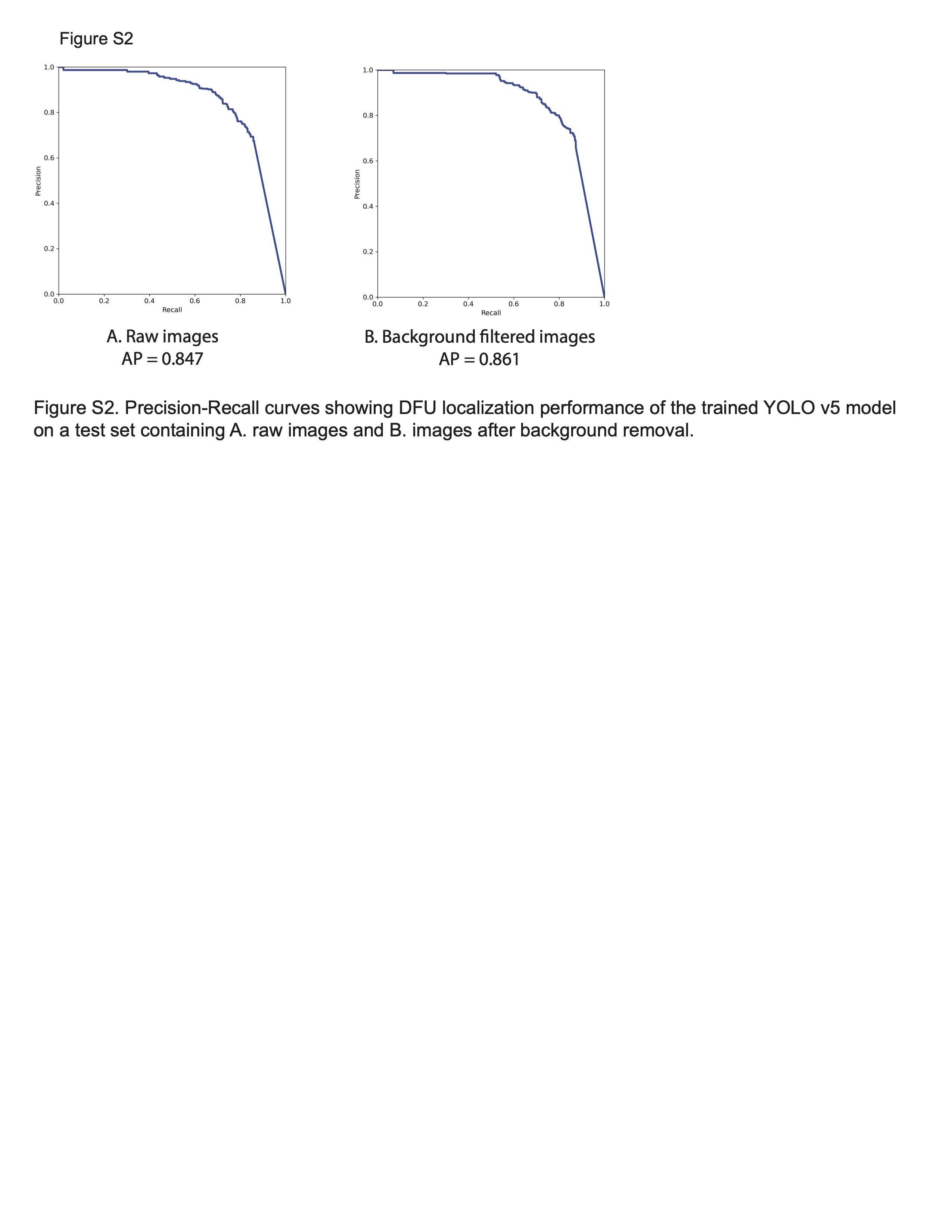

Supplement: Supplementary file 2 [file Image2.jpeg]

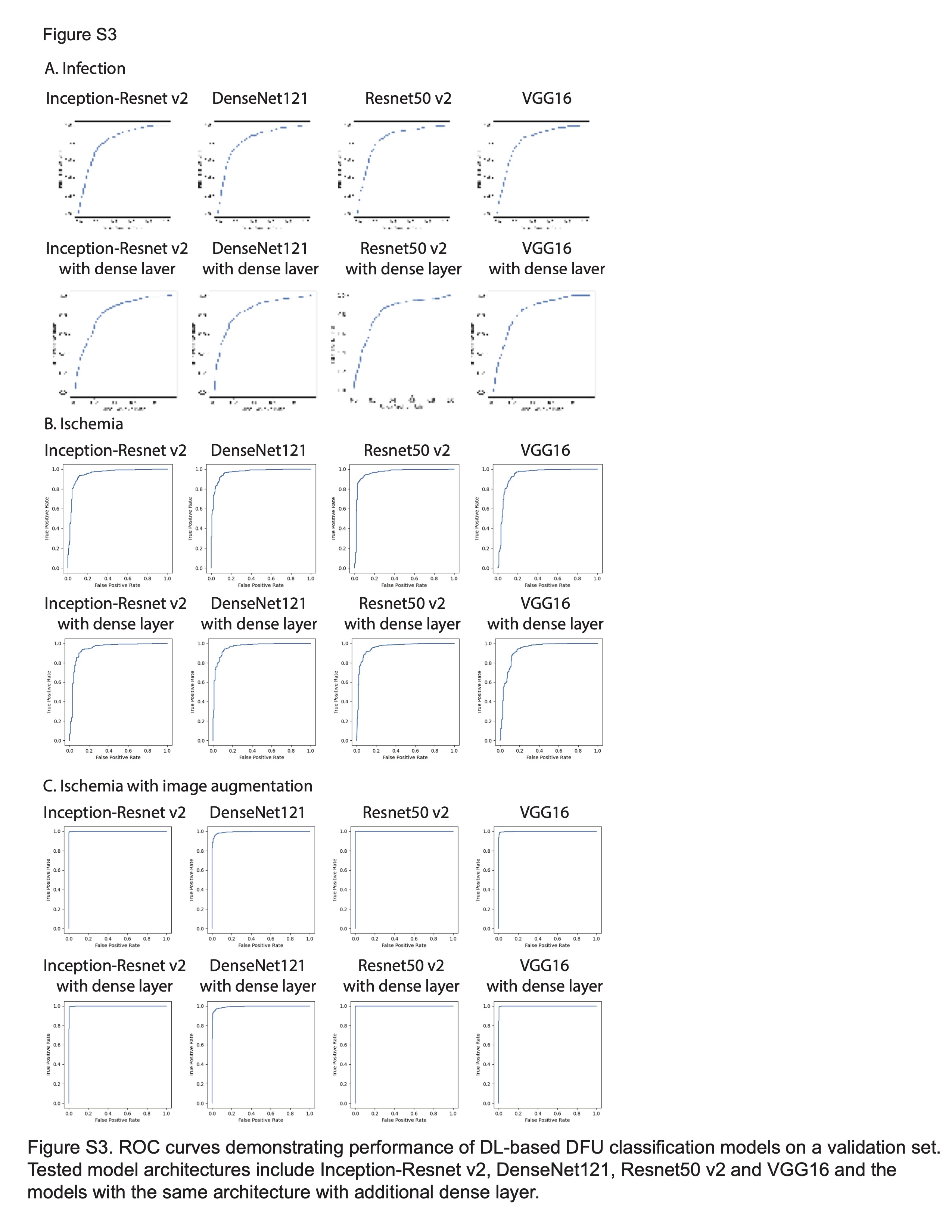

Supplement: Supplementary file 3 [file Image3.jpeg]

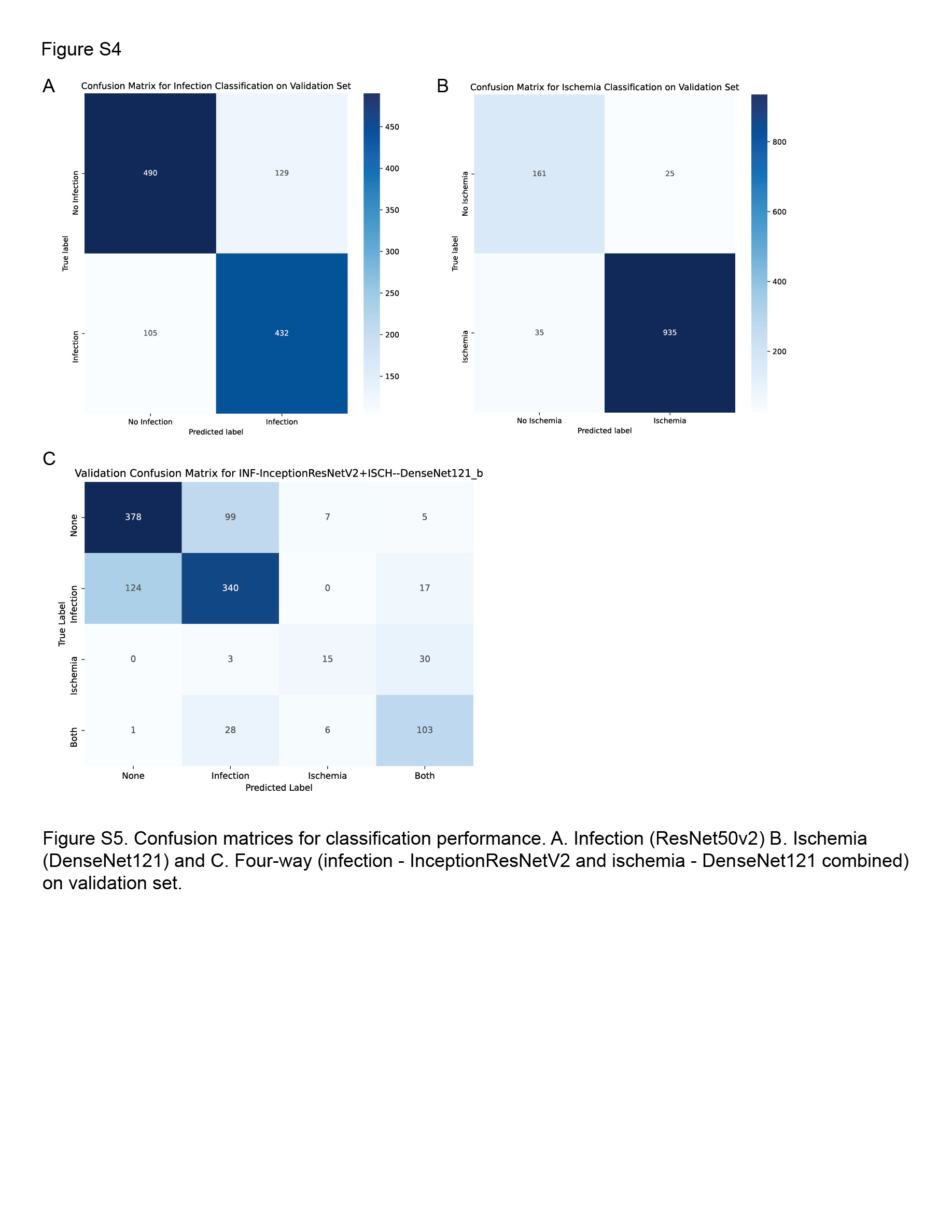

Supplement: Supplementary file 4 [file Image4.jpeg]

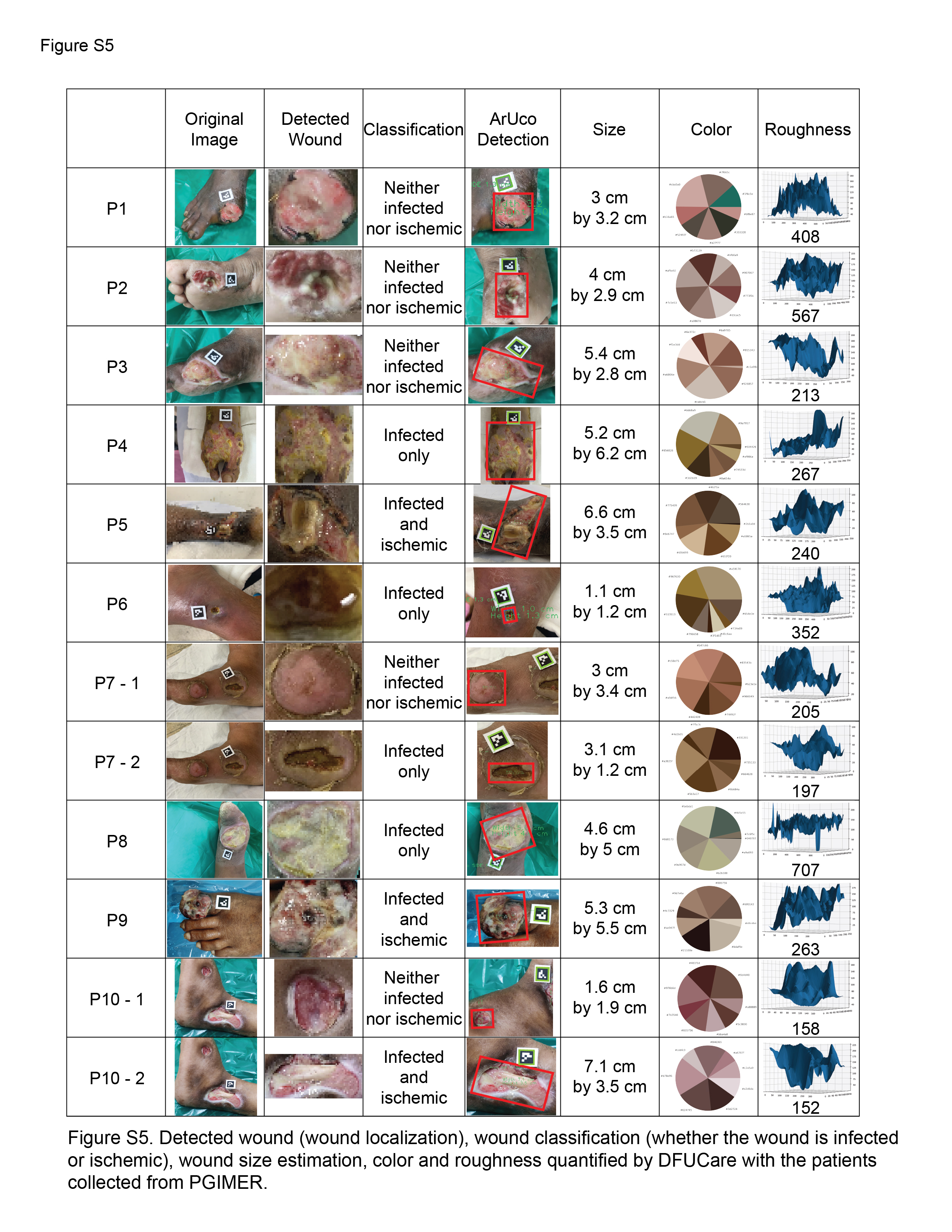

Supplement: Supplementary file 5 [file Image5.jpeg]
