# Supplementary material for: DFUCare: deep learning platform for diabetic foot ulcer detection, analysis, and monitoring
Source: Front Endocrinol (Lausanne). 2024 Sep 23;15:1386613. doi: 10.3389/fendo.2024.1386613 (PMC11460545; doi:10.3389/fendo.2024.1386613)
Supplement: Supplementary file 6 [file Table1.docx]

**Tables**

Table S1. Validation accuracy of DL-based infection and ischemia classification models with different architectures trained and tested on DFUC2020 dataset.

(A) Infection classification

| Model | Accuracy (%) | AUC (%) | Precision (%) | Recall (%) | Specificity (%) |
| --- | --- | --- | --- | --- | --- |
| Inception-ResNet v2 | 76.82 | 82.42 | 75.33 | 74.49 | 78.85 |
| DenseNet121 | 77.34 | 84.07 | 73.27 | 80.63 | 75.12 |
| Resnet50v2 | 79.76 | 84.48 | 76.72 | 81.01 | 74.47 |
| VGG16 | 77.34 | 83.14 | 72.95 | 81.38 | 78.19 |
| Inception-ResNet v2 (with dense layer) | 76.56 | 82.79 | 73.17 | 78.21 | 78.68 |
| DenseNet121  (with dense layer) | 77.77 | 84.64 | 75.45 | 77.28 | 79.16 |
| Resnet50v2  (with dense layer) | 79.76 | 84.9 | 77.01 | 80.45 | 73.83 |
| VGG16  (with dense layer) | 78.2 | 83.42 | 72.44 | 85.66 | 71.73 |

(B) Ischemia classification

| Model | Accuracy (%) | AUC (%) | Precision (%) | Recall (%) | Specificity (%) |
| --- | --- | --- | --- | --- | --- |
| Inception-ResNet v2 | 93.69 | 94.96 | 95.63 | 96.91 | 76.88 |
| DenseNet121 | 94.81 | 96.64 | 97.4 | 96.39 | 74.19 |
| Resnet50v2 | 94.03 | 96.34 | 96.3 | 96.6 | 86.56 |
| VGG16 | 94.55 | 93.97 | 96.13 | 97.42 | 82.80 |
| Inception-ResNet v2 (with dense layer) | 93.86 | 94.75 | 95.18 | 97.63 | 80.65 |
| DenseNet121  (with dense layer) | 94.72 | 96.49 | 96.71 | 97.01 | 82.80 |
| Resnet50v2  (with dense layer) | 93.69 | 94.42 | 96.67 | 95.77 | 79.57 |
| VGG16  (with dense layer) | 93.43 | 91.62 | 95.33 | 96.91 | 75.27 |

(C) Ischemia classification with image augmentation

| Model | Accuracy (%) | AUC (%) | Precision (%) | Recall (%) | Specificity (%) |
| --- | --- | --- | --- | --- | --- |
| Inception-ResNet v2 | 92.65 | 92.64 | 96.63 | 94.54 | 82.80 |
| DenseNet121 | 92.91 | 96.52 | 97.44 | 94.02 | 86.02 |
| Resnet50v2 | 93.34 | 93.62 | 97.25 | 94.74 | 87.10 |
| VGG16 | 93.17 | 92.95 | 96.26 | 95.57 | 87.63 |
| Inception-ResNet v2 (additional layer) | 92.91 | 93.02 | 97.23 | 94.23 | 86.02 |
| DenseNet121  (additional layer) | 92.39 | 96.41 | 97.52 | 93.3 | 83.33 |
| Resnet50v2  (additional layer) | 93.17 | 92.54 | 96.75 | 95.05 | 80.65 |
| VGG16  (additional layer) | 92.65 | 91.34 | 95.76 | 95.46 | 77.96 |

Table S2. Predictive ability of classical machine learnings for infection and ischemia classification trained by handcrafted color and textural feature measured on validation set.

(A) Infection

|  | SVM with RBF kernel | gradBoost after PCA | gradBoost | XGBoost after PCA | XGBoost | MLP |
| --- | --- | --- | --- | --- | --- | --- |
| Accuracy | 0.7725 | 0.8298 | 0.8559 | 0.8344 | 0.8529 | 0.8348 |
| AUC | 0.7731 | 0.7739 | 0.7738 | 0.7853 | 0.7733 | 0.9548 |
| Precision | 0.7603 | 0.8128 | 0.8190 | 0.8197 | 0.8203 | 0.9441 |
| Recall | 0.8035 | 0.8541 | 0.8603 | 0.8572 | 0.8829 | 0.9714 |
| F1 Score | 0.7806 | 0.7736 | 0.7736 | 0.7755 | 0.7737 | 0.9574 |

(B) Ischemia

|  | SVM with RBF kernel | gradBoost after PCA | gradBoost | XGBoost after PCA | XGBoost | MLP |
| --- | --- | --- | --- | --- | --- | --- |
| Accuracy | 0.9289 | 0.9451 | 0.9471 | 0.9542 | 0.9537 | 0.9533 |
| AUC | 0.9159 | 0.8997 | 0.9049 | 0.9286 | 0.9221 | 0.9154 |
| Precision | 0.5902 | 0.8105 | 0.8091 | 0.7930 | 0.8118 | 0.8414 |
| Recall | 0.9001 | 0.8356 | 0.8456 | 0.8945 | 0.7755 | 0.8606 |
| F1 Score | 0.7127 | 0.8233 | 0.8263 | 0.8397 | 0.7815 | 0.8514 |

Table S3. Information of patients collected from Postgraduate Institute of Medical Education and Research (PGIMER) in Chandigarh, India. The number of DFU, whether the wound is infected or ischemic, and the size diagnosed and measured by physician.

|  | Physician Analysis | | |
| --- | --- | --- | --- |
| ID | # of DFU | DFU Classification | DFU size |
| P1 | 1 | Neither infected nor ischemic | 3 cm by 3 cm |
| P2 | 1 | Neither infected nor ischemic | 3 cm by 3 cm |
| P3 | 1 | Neither infected nor ischemic | 5 cm by 3 cm |
| P4 | 1 | Infected only | 5 cm by 6 cm |
| P5 | 1 | Infected only | 7 cm by 3 cm |
| P6 | 1 | Infected only | 1 cm by 1 cm |
| P7 | 2 | 1) Neither infected nor ischemic  2) Infected only | 1) 3 cm by 3 cm  2) 3 cm by 1.5 cm |
| P8 | 1 | Infected only | 5 cm by 5 cm |
| P9 | 1 | Infected and ischemic | 5 cm by 6 cm |
| P10 | 2 | 1) Neither infected nor ischemic  2) Infected and ischemic | 1) 2cm by 2 cm  2) 7 cm by 4 cm |
